# Exploration of Alternative Approaches to Phenotyping of Late Leaf Spot and Groundnut Rosette Virus Disease for Groundnut Breeding

**DOI:** 10.3389/fpls.2022.912332

**Published:** 2022-06-14

**Authors:** Ivan Chapu, David Kalule Okello, Robert C. Ongom Okello, Thomas Lapaka Odong, Sayantan Sarkar, Maria Balota

**Affiliations:** ^1^College of Agricultural and Environmental Sciences, Makerere University, Kampala, Uganda; ^2^National Semi-Arid Resources Research Institute (NaSARRI), Soroti, Uganda; ^3^Blackland Research and Extension Center, Texas A&M AgriLife Research, Temple, TX, United States; ^4^School of Plant and Environmental Sciences, Tidewater AREC, Virginia Tech, Suffolk, VA, United States

**Keywords:** groundnut rosette disease, late leaf spot (LLS), phenotyping, NDVI, RGB indices, logistic models

## Abstract

Late leaf spot (LLS), caused by *Nothopassalora personata* (Berk. & M.A Curt.), and groundnut rosette disease (GRD), [caused by *groundnut rosette virus* (GRV)], represent the most important biotic constraints to groundnut production in Uganda. Application of visual scores in selection for disease resistance presents a challenge especially when breeding experiments are large because it is resource-intensive, subjective, and error-prone. High-throughput phenotyping (HTP) can alleviate these constraints. The objective of this study is to determine if HTP derived indices can replace visual scores in a groundnut breeding program in Uganda. Fifty genotypes were planted under rain-fed conditions at two locations, Nakabango (GRD hotspot) and NaSARRI (LLS hotspot). Three handheld sensors (RGB camera, GreenSeeker, and Thermal camera) were used to collect HTP data on the dates visual scores were taken. Pearson correlation was made between the indices and visual scores, and logistic models for predicting visual scores were developed. Normalized difference vegetation index (NDVI) (*r* = –0.89) and red-green-blue (RGB) color space indices CSI (*r* = 0.76), v* (*r* = –0.80), and b* (*r* = –0.75) were highly correlated with LLS visual scores. NDVI (*r* = –0.72), v* (*r* = –0.71), b* (*r* = –0.64), and GA (*r* = –0.67) were best related to the GRD visual symptoms. Heritability estimates indicated NDVI, green area (GA), greener area (GGA), a*, and hue angle having the highest heritability (*H*^2^ > 0.75). Logistic models developed using these indices were 68% accurate for LLS and 45% accurate for GRD. The accuracy of the models improved to 91 and 84% when the nearest score method was used for LLS and GRD, respectively. Results presented in this study indicated that use of handheld remote sensing tools can improve screening for GRD and LLS resistance, and the best associated indices can be used for indirect selection for resistance and improve genetic gain in groundnut breeding.

## Introduction

Groundnut (*Arachis hypogaea* L.) is the second most important legume in Uganda after the common bean (*Phaseolus vulgaris* L.). It is an important source of protein and vegetable oil and is grown on over 413,000 hectares in Uganda (Deom and Okello, 2018), and over 13 million hectares in Sub-Saharan Africa (SSA; FAOSTAT, 2019). Groundnut productivity in developed countries is higher compared to that in developing countries. For example, the productivity in the USA was 4,072 kg/ha in 2021 (USDA-NASS, 2021), while that in Sub-Saharan Area (SSA) was approximately 950 kg/ha from 2017 to 2019 (FAOSTAT, 2019). The low productivity in SSA is attributed to low agricultural inputs, eroded soil fertility, and extensive biotic stress pressure. Late leaf spot [LLS; caused by *Nothopassalora personata* (Berk. & Curt.) U. Braun, C. Nakash, Videira and Crous] (Giordano et al., 2021) and Groundnut rosette disease (GRD) (caused by *groundnut rosette virus* (GRV)) are the most important biotic constraints to groundnut production in Uganda and SSA (Deom and Okello, 2018). These two diseases often occur simultaneously in farmers’ fields causing up to 100% yield loss depending on the variety and management (Mugisa et al., 2016). Several methods can be employed to control these diseases. For example, GRD can be controlled by early planting and use of optimal plant densities (Farrell, 1976) and spraying insecticides to control the aphid vectors (Davies, 1975; Wightman and Amin, 1988). LLS can be controlled by use of biological control agents, such as chitinolytic bacteria (Kishore et al., 2005) and fungicides (Culbreath et al., 2002). These practices are rarely adopted by smallholder farmers in the SSA because of lack of access to information, shortage of resources, and differential priorities of the crops among different households.

The development of disease-resistant varieties is viewed as the most affordable way for smallholder farmers to maintain stable yields and make economic gains. The development of high-yielding varieties with improved resistance to diseases involves phenotyping of large numbers of breeding lines across multiple breeding locations (Araus and Cairns, 2014). In many breeding programs, early selection for the identification of genotypes with improved performance involves the use of breeder scores based on visual assessment of the plant appearance. For LLS, the breeder scores used in groundnut breeding include a 1–9 severity scale (Subrahmanyam et al., 1995), and for the GRD a 1–5 severity scale (Waliyar et al., 2007). These visual scores are subjective (Milberg et al., 2008), and fully dependent upon the expertise of the evaluator. Therefore, to standardize measurements collected by different evaluators or even the same evaluator at different time points is difficult. Furthermore, visual assessment of plant characteristics in breeding programs is labor-intensive, costly, and time-consuming (Araus and Cairns, 2014) because breeding experiments involve a large number of genotypes planted across multiple locations. Although visual assessment is usually performed by well-trained experts, external factors such as size of plot, time of sampling, and changes in weather conditions can lead to variation in the perception even by the same individual (Borra-Serrano et al., 2018). Visual scores allow the capture of a substantial proportion of variation attributed to genotypic differences, however, methodological inaccuracy usually resulted in low heritability when selection is based on these scores (Visscher et al., 2008). Heritability, along with accuracy and repeatability of the selection method, is important in breeding because traits with high value of heritability are more likely to significantly contribute to the genetic gain (Cobb et al., 2019). A high heritability is indicative of great contribution of genetic factors compared to environmental factors to the expression of a trait (Holland et al., 2003). Several studies have reported high heritability of visual scores of LLS (Anderson et al., 1991) and GRD (Merwe et al., 1999) indicating possibility of attaining genetic gains using these scores. Alternative methods, which involve the use of proxy traits, such as canopy temperature and NDVI, have registered improved heritability, better selection accuracy, and higher genetic gains in various crops, such as sugar cane (*Saccharum officinarum* L.) (Natarajan et al., 2019), cotton (*Gossypium hirsutum* L.) (Andrade-Sanchez et al., 2013), and wheat (*Triticum aestivum* L.) (Wang et al., 2020).

High-throughput phenotyping (HTP) platforms have the potential to ameliorate the challenges associated with visual assessments and, consequently, accelerate genetic gain (Araus and Cairns, 2014; Araus Ortega et al. 2014, 2018). HTP involves the use of advanced technologies for fast data collection and processing, and non-destructive and non-invasive analysis of plant characteristics (Gehan and Kellogg, 2017). HTP platforms offer detailed measurements of plant characteristics of interest (Finkel, 2009). Previous studies have demonstrated the efficacy of the RGB imaging for assessment of yellow rust (*Puccinia striiformis f.* sp. tritici) in wheat (*Triticum aestivum* L.) (Zaman-Allah et al., 2015; Zhou et al., 2015), Verticillium wilt (caused by *Verticillium dahliae* Kleb) in olive (*Olea europaea* L.) (Sancho-Adamson et al., 2019), and lethal necrosis [caused by a combination of *maize chlorotic mottle virus* (MCMV) and *sugar cane mosaic virus* (SCMV)] in maize (*Zea mays* L.) (Kefauver et al., 2015). NDVI has widely been applied in breeding for resistance to yellow rust in wheat and maize, lethal necrosis in maize (Kefauver et al., 2015), and powdery mildew (*Blumeria graminis* f. sp. tritici) in wheat (Franke and Menz, 2007). Canopy temperature has been applied for stripe rust phenotyping in wheat (Cheng et al., 2015), and downey mildew (*Pseudoperonospora cubensis* (Berk & M.A Curtis) Rostovzev) in cucumber (*Cucumis sativis* L.) (Oerke et al., 2006) and tomato (*Solanum lycopersicum* L.) (Raza et al., 2015).

In groundnut breeding, application of HTP methods to complement or replace traditional phenotyping is in incipient stages. Efforts have been put forward to develop HTP methods to assess leaf wilting (Sarkar et al., 2021), plant height (Yuan et al., 2019; Sarkar et al., 2020), and plant population and variety differentiation using RGB and NDVI (Oakes and Balota, 2017). No HTP methods are yet available for phenotyping LLS and GRD resistance in groundnut. The changes in plant physiology and morphology under LLS and GRD pressure can be phenotyped remotely and genotypic differences for resistance to these diseases can be assessed, as in other crops. In this study, handheld tools were used to develop HTP methods to improve phenotyping accuracy within groundnut breeding programs in Uganda and SSA. The overall objective of this study was to evaluate the effectiveness of several handheld sensors as low-cost phenotyping tools for screening LLS and GRD resistance in groundnut breeding. Specific objectives were as follows: (1) To evaluate the relationship between sensor-derived vegetation indices (VIs) with the LLS and GRD conventional visual scores; (2) determine the heritability of the VIs at different groundnut growth stages; and (3) develop VI-based regression models for selection for LLS and GRD resistance in groundnut breeding programs.

## Materials and Methods

### Field Experiment

The experiment was conducted during two planting seasons: 2020A (April–August 2020) and 2020B (September–December 2020), at the National Semi-Arid Recourses Research Institute (NaSARRI), Serere District, Eastern Uganda (1°0′00.0″N, 33°33′00.0″E), and Nakabango Technology Verification Center, Jinja District, Eastern Uganda (0°31′17.6″N, 33°12′49.1″E). These locations receive bimodal rainfall seasons throughout the year; the first between March and May, and the second between August and October. These locations were chosen because they were characterized as hot spots for both GRD (Nakabango) and LLS (Nakabango and NaSARRI) screening in Uganda (Okello et al., 2010). Breeding genotypes were selected for this study; 50 genotypes consisting of commercial varieties and advanced breeding lines from NaSARRI, Uganda, and International Crop Research Institute for the Semi-Arid Tropics (ICRISAT), Malawi. This selected population included three groundnut market types: Virginia (bv. *hypogaea*), Spanish (bv. *vulgaris*), and Valencia (bv. *fastigiata*). The 50 genotypes were selected to represent the different levels of resistance to GRD and LLS as shown in (Table 1). The experiment was laid out in 5 x 10 alpha-lattice design with three replications. The genotypes were planted in two-row plots of 1-m long × 45 m wide. The experimental layout was generated from the Breeding Management Systems (BMS) platform [The IBP Breeding Management System (BMS Pro) Version 13 (2020)].^1^ The spacing between the plots within the blocks was 0.6 m, 0.45 m between rows within the plot, and 0.15 m between plants within the row. A distance of 0.9 m was kept between the blocks and 1.5 m between the replicates. The experiment was maintained under rain-fed conditions and standard agronomic practices (Okello et al., 2010).

**TABLE 1:** A list of genotypes used in the study showing their market type, source, and status.

Entry	Genotype	Market type	Source	Status
1	ICGV-SM 03590	Spanish	ICRISAT	Resistant check LLS
2	DOK 1R	Spanish	NaSARRI	Resistant check; GRD
3	Serenut 14R	Virginia	NaSARRI	Resistant check; LLS & GRD
4	Serenut 7T	Virginia	NaSARRI	Resistant check; LLS & GRD
5	Serenut 11T	Virginia	NaSARRI	Resistant check; LLS & GRD
6	Serenut 8R	Virginia	NaSARRI	Resistant check; LLS & GRD
7	Serenut 9T	Virginia	NaSARRI	Resistant check; LLS & GRD
8	Serenut 4T	Spanish	NaSARRI	Susceptible check; LLS
9	Serenut 6T	Spanish	NaSARRI	Susceptible check; LLS
10	JL 24	Spanish	ICRISAT	Susceptible check; LLS & GRD
11	Acholi white	Valencia	NaSARRI	Susceptible check; LLS & GRD
12	RedBeauty	Valencia	NaSARRI	Susceptible check; LLS & GRD
13	SGV 10010 ER	Spanish	NaSARRI	Test entry
14	DOK 1T	Spanish	NaSARRI	Test entry
15	ICGV 02501	Spanish	ICRISAT	Test entry
16	SGV 0080	Virginia	NaSARRI	Test entry
17	ICGV-SM 16502	Spanish	ICRISAT	Test entry
18	SGV 0060	Virginia	NaSARRI	Test entry
19	SGV 0075	Virginia	NaSARRI	Test entry
20	ICGV 01502	Spanish	ICRISAT	Test entry
21	SGV 07010	Virginia	NaSARRI	Test entry
22	Serenut 5R	Virginia	NaSARRI	Test entry
23	12CS-015	Spanish	USA-UGA	Test entry
24	B7-30-9-3	Spanish	USA-UGA	Test entry
25	ICGV-SM 08556	Spanish	ICRISAT	Test entry
26	ICGV-SM 01709	Virginia	ICRISAT	Test entry
27	ICGV-SM 95526	Valencia	ICRISAT	Test entry
28	ICGV-SM 95355	Virginia	ICRISAT	Test entry
29	ICGV-SM 16520	Spanish	ICRISAT	Test entry
30	ICGV 01504	Spanish	ICRISAT	Test entry
31	SGV 0805	Valencia	NaSARRI	Test entry
32	ICGV-SM 01731	Virginia	ICRISAT	Test entry
33	ICGV-SM 95714	Valencia	ICRISAT	Test entry
34	ICGV-SM 99568	Spanish	ICRISAT	Test entry
35	ICGV-SM 88710	Virginia	ICRISAT	Test entry
36	SGV 0071	Virginia	NaSARRI	Test entry
37	SGV 0084	Virginia	NaSARRI	Test entry
38	ICGV 01514	Spanish	ICRISAT	Test entry
39	ICGV-SM 03702	Virginia	ICRISAT	Test entry
40	SGV 0023	Virginia	NaSARRI	Test entry
41	SGV 990400	Virginia	NaSARRI	Test entry
42	ICGV-SM 16501	Spanish	ICRISAT	Test entry
43	ICGV-SM 0205	Spanish	ICRISAT	Test entry
44	ICGV 9555	Spanish	ICRISAT	Test entry
45	SGV 10005	Spanish	NaSARRI	Test entry
46	ICGV 01515	Spanish	ICRISAT	Test entry
47	ICGV 01510	Spanish	ICRISAT	Test entry
48	ICGV-SM 96714	Valencia	ICRISAT	Test entry
49	SGV 0065	Virginia	NaSARRI	Test entry
50	SGV 0047	Virginia	NaSARRI	Test entry

### Traditional Visual Assessment/Scoring

Visual scores of disease severity were taken by a groundnut breeding technician. Disease data was collected four times across the growing season each data point corresponding with a particular phenological stage of the groundnut. The data was collected at growth stages R2 (beginning of peg formation), R4 (beginning of pod formation), R7 (beginning of maturity), and R8 (harvest maturity) as described by Boote (1982). LLS severity was scored based on a 1–9 visual scale as described in Table 2 (Subrahmanyam et al., 1995). GRD was scored according to Equation 1 based on percentage disease incidence at beginning of peg formation, beginning of pod formation, beginning of maturity and GRD severity at harvest maturity. GRD severity was scored based on a 1–5 scale (Table 3; Waliyar et al., 2007).


(1) $$GRDIncidence\left(\%\right)=\frac{N\,u\,m\,b\,e\,r\,o\,f\,i\,n\,f\,e\,c\,t\,e\,d\,p\,l\,a\,n\,t\,s}{T\,o\,t\,a\,l\,n\,u\,m\,b\,e\,r\,o\,f\,p\,l\,a\,n\,t\,s}\times 100\%$$

**TABLE 2:** The 9 point scale used for screening groundnut for resistance for late leaf spot (Subrahmanyam et al., 1995).

Score	Description	Disease severity (%)	Inference
1	No disease	0	Resistant
2	Lesions present on lower leaves; no defoliation	1–5	Resistant
3	Lesions present largely on lower leaves, very few on middle leaves; defoliation of some leaflets on lower leaves	6–10	Resistant
4	Lesions on lower and middle leaves but severe on lower leaves, defoliation of some leaflets evident on lower leaves	11–20	Moderately resistant
5	Lesions present on all lower and middle leaves; over 50% defoliation of lower leaves	21–30	Moderately resistant
6	Severe lesions on lower and middle leaves; lesions present but less severe on top leaves; extensive defoliation of lower leaves; defoliation of some leaflets evident on middle leaves	31–40	Moderately resistant
7	Lesions on all leaves but less severe on top leaves; defoliation of all lower and some middle leaves	4–60	Susceptible
8	Defoliation of all lower and middle leaves; severe lesions on top leaves; some defoliation of top leaves evident	61–80	Susceptible
9	Almost all leaves defoliated, leaving bare stems; some leaflets may remain, but show severe leaf spots	81–100	Susceptible

**TABLE 3:** Groundnut rosette severity scale as described by Waliyar et al. (2007).

Score	Genotype reaction	Inference
1	No visible symptoms on the foliage	Highly resistant
2	Rosette symptoms on 1–20% foliage, but no obvious stunting	Resistant
3	Rosette symptoms on 21–50% foliage and stunting	Moderately resistant
4	Severe rosette symptoms on 51–70% foliage and stunting	Susceptible
5	Severe symptoms on 71–100% foliage, stunted or dead plants	Highly susceptible

### High-Throughput Measurements

**FIGURE 1:**
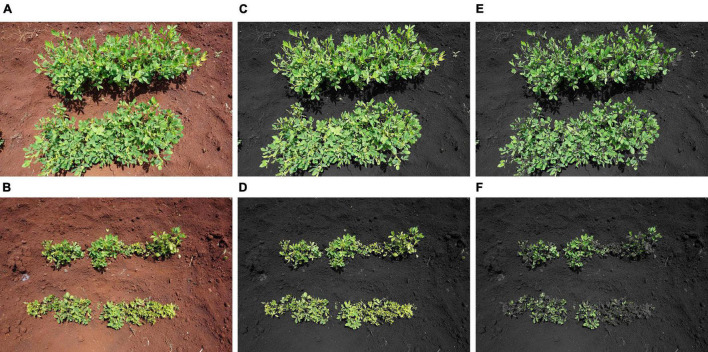
RGB images of groundnut rosette disease resistant **(A)** and susceptible **(B)** genotypes taken at 0.8 m above the plant canopy before and after analysis using BreedPix 0.2 option of the maize scanner 1.6. Images A and B show the original images, images **(C,D)** show the green area (GA), and images **(E,F)** show the greener area (GGA) with all yellowish (hue angle 60–80°) pixels removed.

A Sony α6000 digital camera [model ILCE 6000, 24.3 megapixel (Sony-α, Tokyo, Japan)], GreenSeeker crop sensor (Trimble Inc., Sunnyvale, California, United States), and FLIR C2 Thermal camera (Teledyne FLIR LCC, Wilsonvile, Oregon, United States) were used as HTP sensors in this study. HTP measurements were taken on the same dates as traditional visual scoring. The HTP measurements were taken between 10:00 and 16:00 h, on sunny days. To collect the RGB images, the Sony α6000 camera was set to auto so that the lens could adjust to the best sharpness, brightness, and hue based on available light. The camera zoom was set at 0 for all images and a 58 mm camera lens was used. The camera was held at 90 cm above the plant canopy in a zenithal plane and focusing at the center of each plot. The camera had an F-stop of f/8.0, a focal length of 16 mm, and an ISO speed ISO-100 without a flash. The images were saved as Joint Photographic Experts Group (JPEG) files (6,000 × 4,000) with a resolution of 350 dpi. RGB color space indices were extracted from the images using the BreedPix 0.2 option of the CIMMYT maize scanner 1.6 plugin (open software^2^; Copyright 2015 Shawn Carlisle Kefauver, University of Barcelona; produced as part of Image J/Fiji (open source software)^3^ (Schindelin et al., 2012; Rueden et al., 2017). Figure 1 illustrates the extraction of the RGB indices using BreedPix, while Table 4 presents the indices and their description.

**TABLE 4:** RGB indices derived from the BreedPix and their description.

RGB Indices	Basis of derivation	Color space	References
Hue	Color description in form of angles [0°–360° (0°-red; 60°-yellow; 120°-green; 240°-blue)]	His	Cheng et al., 2001
Saturation	Measure of dilution of pure hue with white light [0–1]	HSI	
Intensity	Measure of grayness on a 0 (black)–1 (white) scale	HSI	
Lightness	Light reflected by a non-luminous body [0 (black)–100 (white scale)]	CIE-Lab	
a*	Green (a)-Red ( + a) component	CIE-Lab	Cheng et al., 2001
b*	Blue (–b)-Yellow ( + b) component	CIE-Lab	
u*	Green (–u)-Red ( + u) component	CIE-Luv	
v*	Blue (–v)-Yellow ( + v) component	CIE-Luv	
Green area (GA)	Pixels from 60°–120°	HIS	Casadesus et al., 2007; Kefauver et al., 2015; Zhou et al., 2015
Greener area (GGA)	Pixels from 80°–120°	HIS	
Crop senescence index (CSI)	100×(*GA*−*GGA*)/*GA*	HIS	Zaman-Allah et al., 2015
ab	*a* * *b* *	CIE-Lab	
Normalized difference CIE-lab index (NDLab)	$\frac{1-a^{*}-b^{*}}{1-a^{*}+b^{*}}+1$	CIE-Lab	Buchaillot et al., 2019
uv	*u* * *v* *	CIE-Luv	
Normalized difference CIE-luv index (NDLuv)	$\frac{1-u^{*}-v^{*}}{1-u^{*}+v^{*}}+1$	CIE-Luv	Buchaillot et al., 2019

*In this case is associated with the CIE-Lab indices a and b and CIE-Luv indices u and v as derived from the BreedPix.

Canopy NDVI values were determined using a handheld spectroradiometer (GreenSeeker crop sensor, Trimble United States) on the same date the RGB images were taken. The GreenSeeker was held at 60 cm above the plant canopy and average NDVI readings were taken from each row. The trigger of the GreenSeeker was pressed at the beginning of the row and released at the end of the row to obtain the average NDVI of the row. The average of the two rows was taken to determine the plot average NDVI reading. The readings were taken when the sun was overhead to avoid shadows. NDVI was calculated according to Equation 2.


(2) N⁢D⁢V⁢I=((N⁢I⁢R-R))/((N⁢I⁢R+R))

Where *R* is the reflectance in the red band (660 nm) and NIR is the reflectance in the reflectance in the near-infrared band (760 nm).

**FIGURE 2:**
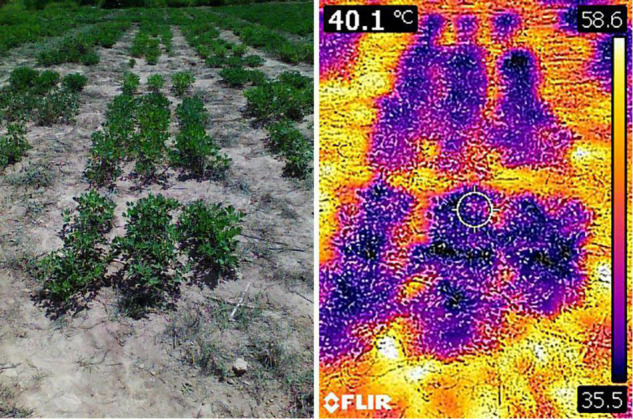
RGB image of selected plot taken using FLIR C2 camera **(left side)** and corresponding thermal image **(right side)** as seen in FLIR Tools software.

Canopy temperature (CT) was measured using a FLIR C2 Thermal camera with a focal length of 2 mm. Images were taken while holding the camera 60–80 cm from the plant at an angle of 45°, and the images were saved as JPEG files with dimensions of 240 × 320 pixels and a resolution of 72 dpi. FLIR Tools software was used to extract the canopy temperature readings from the thermal images in degrees centigrade (Figure 2).

### Statistical Analysis

#### Pearson Correlation of Visual Scores and Vegetation Indices

The means of visual measurements and HTP-derived VIs of each genotype were extracted for each day of data collection using the R statistical software (R Core Team, 2021). Pearson correlation was performed between the means of the visual scores and VIs taken at the same time point and for all data points combined, and for individual market types and all the market types combined using the *rcorr* function of the *Hmisc* package of the R software.

#### Calculation of Broad Sense Heritability

The broad-sense heritability (*H*^2^*)* was calculated as according to Equation 3


(3) $$H^{2}=\frac{\sigma_{G}^{2}}{\sigma_{G}^{2}+\frac{\sigma_{G\,E}^{2}}{n}+\frac{\sigma_{e}^{2}}{n\,r}}$$

Where $\sigma_{g}^{2}$ is the genotypic variance, $\sigma_{G\,E}^{2}$is the variance of the genotype by environment (location) interaction, $\sigma_{e}^{2}$ is the residual error variance, n is the number of environments (locations) and r is the number of replications within each environment (Piepho and Mohring, 2007). The variance components used were estimated from the analysis of variance (ANOVA) using the *lmer* function of the *lme4* package (Bates et al., 2015) of the R statistical software (R Core Team, 2021).

### Development of Regression Models

#### Training of Regression Models

Ordinal logistic regression was used to develop two separate prediction models, one for the LLS and one for GRD, disease severity. The models were developed from data collected at R8 (harvest maturity) because the strongest association between VIs and visual scores was recorded at maturity. Ordinal logistic regression was applied in this case because the GRD and LLS scales used in the study were ordinal variables. The NDVI readings, RGB, and thermal images taken per plot together with the visual scores allocated to the respective plots were all used in the models. The regression models were developed using stepwise regression using the *caret* (Classification And Regression Training) package (Max et al., 2021) and the “*polr*” function of the MASS package of R. Data from Nakabango for the 2020A and 2020B seasons was used for training the ordinal logistic regression models and data from NaSARRI was used to validate the models. The backward selection method of stepwise regression was used to select the parameters with the highest contribution to the model. A full model (all predictors) was fitted and the least contributing predictors were removed until all the parameters in the model were statistically significant (*P* < 0.05). K-fold cross-validation was used to evaluate the models. The training data set was randomly split into *k*-folds (*k* = 10). The model was trained on nine subsets and one subset was reserved for testing the model. The process was repeated until each of the k-subsets has served as the testing set. The ten sets of results were then averaged to produce a single model estimation. Akaike’s Information Criteria (AIC) and Bayesian Information Criteria (BIC) were used to select the best model. Models with lower AIC and BIC are better predictors than those with higher values. The accuracy of the models was derived from the classification accuracy matrices (Equation 4).


(4) $$A\,c\,c\,u\,r\,a\,c\,y=\frac{N\,o.o\,f\,p\,l\,o\,t\,s\,c\,l\,a\,s\,s\,i\,f\,i\,e\,d\,c\,o\,r\,r\,e\,c\,t\,l\,y}{T\,o\,t\,a\,l\,p\,l\,o\,t\,s\,i\,n\,t\,h\,e\,s\,e\,t}\times 100$$

The nearest score method was also used to improve the classification accuracy of the models. The visually rated scores were matched with the model derived scores, and two of the nearest scores (preceding and succeeding) of the visually rated scores were also matched. If any of the values (actual values, preceding or succeeding) matched with the model derived score, it was assumed as the correct classification (Sarkar et al., 2021).

#### Validation of Regression Models

The regression models developed using data from Nakabango were validated using data from NaSARRI. The model was applied to the validation data set to obtain predicted values. A confusion matrix (Sarkar et al., 2021) was developed to determine the accuracy of the model.

## Results

### Distribution of High-Throughput Phenotyping Measurements and Visual Scores

The data presented was collected in Nakabango across the two growing seasons; 2020A and 2020B. The disease pressure was higher in Nakabango across the two seasons. The distribution of the LLS visual scores increased over time peaking at harvest maturity which coincided with the time of harvest. The Spanish and Valencia market types had similar patterns of distribution of LLS scores over time. At all-time points, LLS scores were higher for Valencia and Spanish type compared to Virginia (Virginia appear more resistant). At each data collection time, more variability in LLS score was registered in Valencia and Spanish types compared to Virginia. Similarly, for GRD visual scores, the Spanish and Valencia market types showed a similar pattern of distribution over time. The variability in GRD visual symptoms was higher for the Spanish and Valencia, and lower for the Virginia market type at all-time points.

**FIGURE 3:**
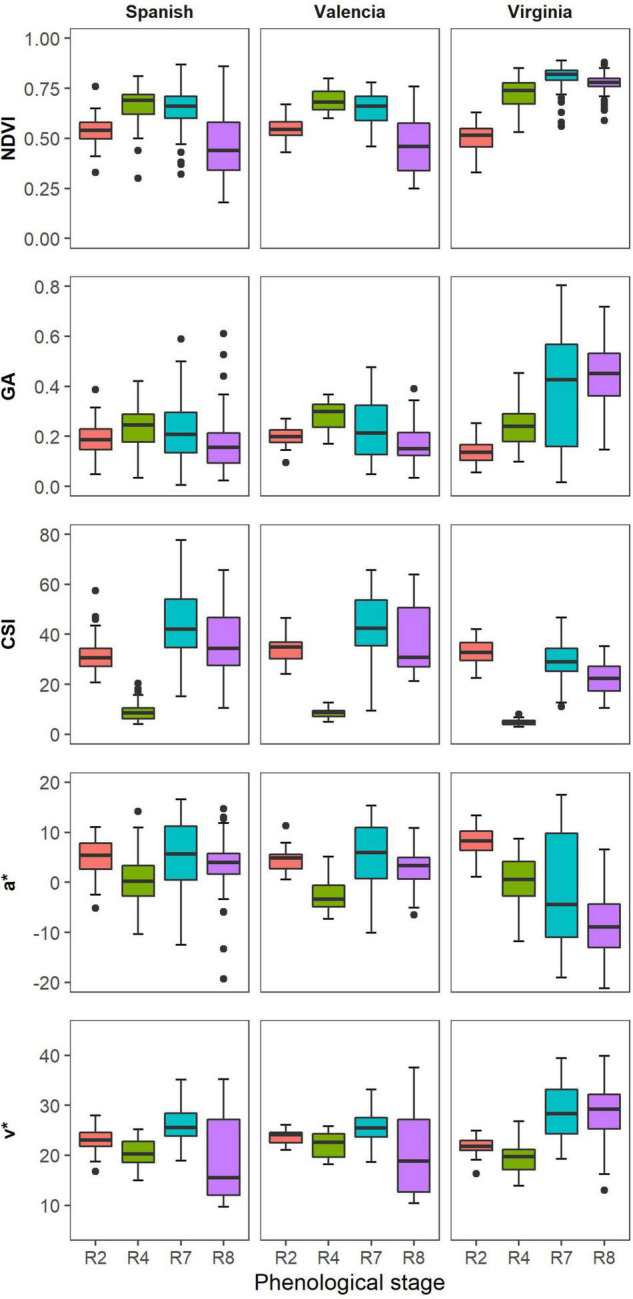
Box plots illustrate the distribution of selected VIs of the different market types across four time points during the growth season.

Valencia and Spanish market types showed similarities in the distribution of vegetation indices (VIs) indices with Virginia showing different responses. The NDVI values were similar for all the market types at beginning of peg formation (Figure 3). At full pod formation (R4), all market types had similar medians for the GA, but the variability was greater for Spanish compared to Valencia and Virginia. At beginning of maturity (R7) and harvest maturity (R8), Virginia had higher GA values compared to the other two market types, just like for the NDVI. Within each market type, the CSI medians were lowest at full pod formation (R4) and highest at beginning of maturity, but Virginia market types had lower CSI medians in comparison with Spanish and Valencia at R7. The spread of the CSI values was highest at beginning of maturity for all the market types, but higher for the Spanish and Valencia compared to Virginia. The distributions of the medians of a* and u* followed similar trends for all market types. The medians were highest at beginning of pegging and lowest at full pod formation. The spread of measurements was highest at beginning of maturity and lowest at beginning of peg formation.

### Pearson Correlation Between Visual Scores and Vegetation Indices

**FIGURE 4:**
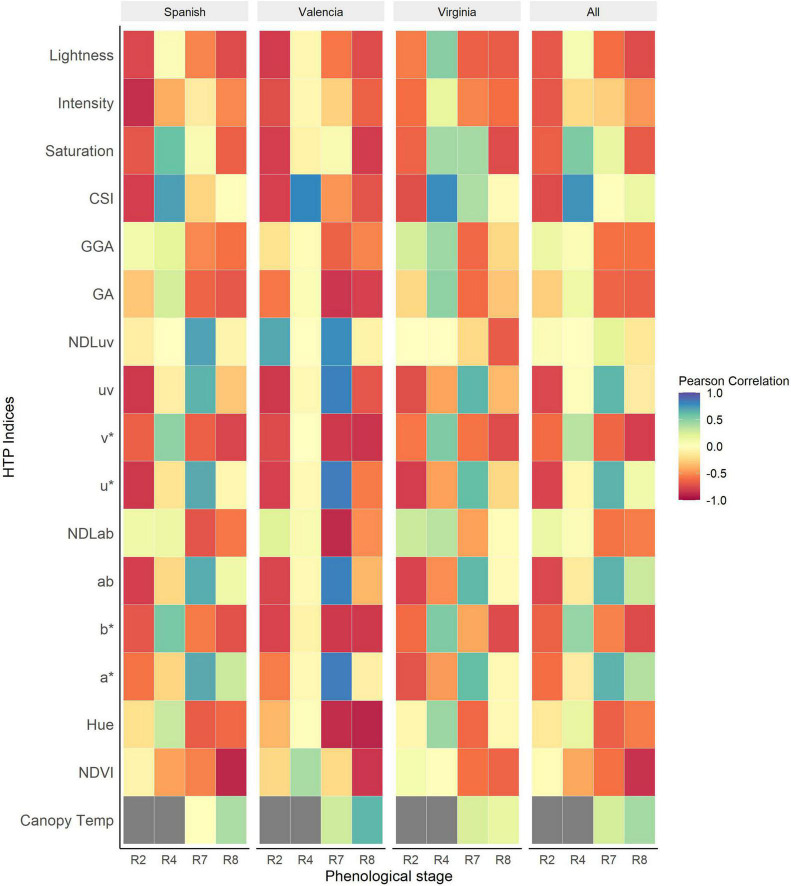
Heatmap of Pearson correlations between HTP derived indices and visual scores of Late leaf spot (LLS) severity at different phenological stages of the growth season for the individual market types and all the market types analyzed together. The dark gray boxes represent missing data.

The Pearson correlation between visual scores and the vegetation HTP indices (VIs) was performed using different subsets of the data (each market type, collection dates and for the combined dataset of the three market types). The strongest correlations between LLS visual scores and VIs were recorded at R2 (beginning of pegging) and R8 (harvest maturity). The association between the VIs and NDVI show an increase throughout the season peaking at R8 (*r* = –0.85, *P* < 0.001) (Figure 4). The NDVI was strongly associated with LLS severity of all market types at harvest maturity, with the strongest association recorded in the Spanish type (*r* = –0.89, *P* < 0.001). For Valencia, there was non-significant association between LLS visual score and NDVI until harvest maturity (*r* = –0.83, *P* < 0.001) and for the Virginia type, the association was non-significant as well until harvest maturity (*r* = –0.64, *P* < 0.001).

RGB indices were significantly correlated with the disease symptoms of all the three market types at harvest [Lightness (*r* = –0.74, *P* < 0.001), v* (*r* = –0.80, *P* < 0.001), and b* (*r* = –0.75, *P* < 0.001)]. At R4 (full pod), CSI (*r* = 0.76, *P* < 0.001) was the most sensitive index to the onset of LLS. Some indices, however, performed better only for some market types at particular time points. For example, the RGB indices Hue (*r* = –0.87, *P* < 0.001), a* (*r* = 0.84, *P* < 0.001), b* (*r* = –0.82, *P* < 0.001), NDLab (*r* = –0.88, *P* < 0.001), u* (*r* = 0.84, *P* < 0.001), v* (*r* = –0.82, *P* < 0.001), and GA (*r* = –0.83, *P* < 0.001) were highly correlated with disease symptoms of Valencia market type at R7 (beginning of maturity) but moderately correlated with Virginia and Spanish market type LLS symptoms. At full pod (R4), RGB indices Hue, Saturation, b*, v*, and GA were significantly associated with disease symptoms of Virginia (*r* = 0.45, 0.43, 0.53, 0.53, 0.48; *P* < 0.001) and Spanish (*r* = 0.30, 0.59, 0.55, 0.53, 0.26; *P* < 0.001) market types but insignificant for the Valencia type. Canopy temperature (*r* = 0.42, *P* < 0.001) was positively correlated with disease severity, of all the three market types across the different time points. The correlation of the Valencia type (*r* = 0.64, *P* < 0.001) was stronger compared to that of the Spanish (*r* = 0.4, *P* < 0.001) and Virginia (*r* = 0.16, *P* < 0.001) market types.

The association between NDVI and the GRD scores show a gradual increase over the season peaking at beginning of maturity. The association between the visual scores and NDVI was strongest at beginning of maturity (*r* = –0.73, *P* < 0.001) and full maturity (*r = –*0.72, *P* < 0.001). The associations among the Spanish and Valencia market types were stronger compared to those among the Virginia type at all data collection points.

**FIGURE 5:**
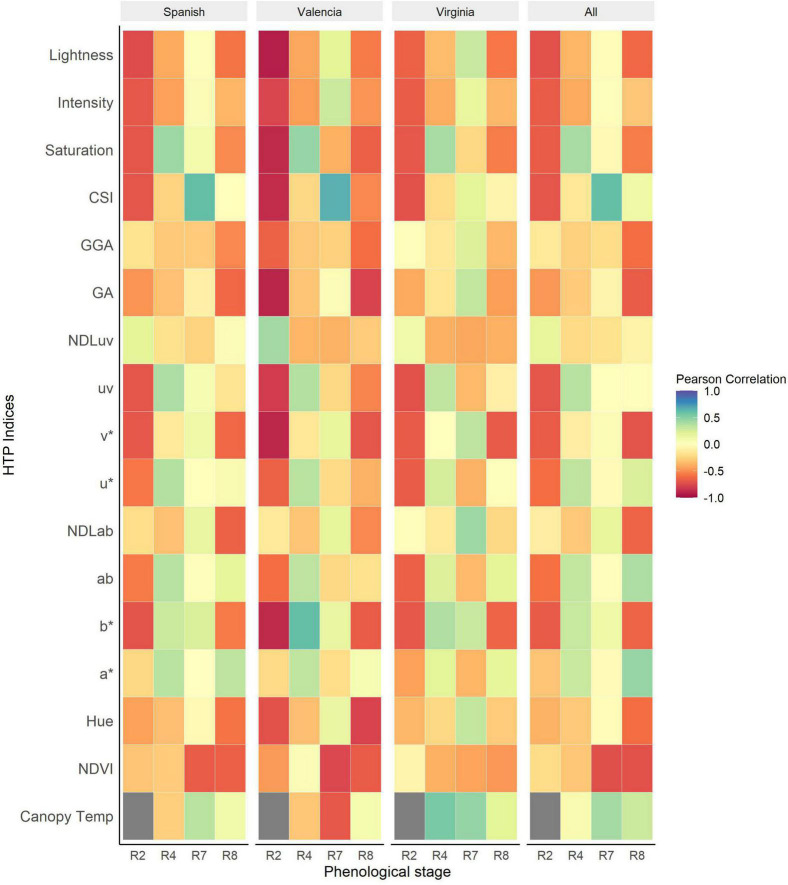
Heatmap of Pearson correlations between HTP derived indices and visual scores of Groundnut rosette disease (GRD) at different phenological stages of the growing season for the individual market types and all the market types analyzed together. The dark gray boxes represent missing data.

The RGB indices were significantly associated with GRD visual scores at beginning of peg formation and at harvest maturity for all the market types. The strongest associations were recorded at harvest maturity [v* (*r* = –0.71, *P* < 0.001), GA (*r* = –0.67, *P* < 0.001), NDLab (*r* = –0.64, *P* < 0.001), GGA (*r* = –0.6, *P* < 0.001), Lightness (*r* = –0.62, *P* < 0.001)]. For CSI, the associations gradually increased throughout the season from (*r = –*0.7, *P* < 0.001) beginning of peg formation (R2) and peaking at beginning of maturity (R7) (*r* = 0.6, *P* < 0.001 (Figure 5). The associations were generally higher for the Valencia type compared to the Spanish and Virginia type. Early (R2) detection of disease symptoms was possible among the Valencia market type using RGB indices saturation, lightness (*r = –*0.92, *P* < 0.001), b* (*r* = –0.88, *P* < 0.001), v* (*r = –*0.89, *P* < 0.001), GA (*r = –*0.90, *P* < 0.001), and CSI (*r* = 0.87, *P* < 0.001). Canopy temperature was generally positively correlated with disease symptoms for all market types. The associations were highest at beginning of maturity (*r* = 0.42, *P* < 0.001) and non-significant at full pod formation and harvest maturity. At full pod formation (R4), the Valencia and Spanish had a negative association compared to the Virginia type with positive association. However, at beginning of maturity, the Spanish (*r* = 0.35, *P* < 0.001) and Virginia (*r* = 0.47, *P* < 0.001) had a similar trend with positive correlations compared to Valencia (*r = –*0.69, *P* < 0.001) market type with a negative association.

### Identification of Resistant and Susceptible Genotypes

Identification of the most resistant and susceptible genotypes based on the visual and NDVI rankings is presented in Table 5. For LLS, NDVI identified three of the top five most resistant genotypes based on the visual score ranking. However, both NDVI and visual scores each identified different five, most susceptible, genotypes within the population. For GRD, both NDVI and visual scores identified three common genotypes out of the five most resistant and susceptible genotypes (Table 5).

**TABLE 5:** Table genotype ranking for LLS and GRD using visual scores and NDVI. The genotype ranking was done using genotype means.

	Late leaf spot		Groundnut rosette disease	
Rank	Visual score	NDVI	Visual score	NDVI
1	ICGV-SM 03590^a^	ICGV-SM 03590^a^	DOK 1R^a^	ICGV-SM 03590^a^
2	Serenut 14R^a^	Serenut 14R^a^	Serenut 14R^a^	Serenut 14R^a^
3	SGV 990400	Serenut 8R^a^	Serenut 8R^a^	Serenut 8R^a^
4	Serenut 9T^a^	Serenut 9T^a^	Serenut 9T^a^	Serenut 9T^a^
5	SGV 0071	SGV 0060	ICGV-SM 01709	SGV 0060
46	ICGV-SM 08556	Acholi White^b^	JL 24^b^	Acholi White^b^
47	ICGV-SM16520	ICGV-SM 16501	RedBeauty^b^	ICGV-SM 16501
48	ICGV 01504	ICGV-SM 96714	ICGV-SM 96714	ICGV-SM 96714
49	ICGV-SM 16502	RedBeauty^b^	ICGV-SM 0205	RedBeauty^b^
50	Serenut 4T^a^	SGV 10005	Acholi White^b^	SGV 10005

*^a^Resistant check*, ^b^susceptible check.

*In this case is associated with the CIE-Lab indices a and b and CIE-Luv indices u and v as derived from the BreedPix.

### Heritability

Table 6 shows the *H*^2^ values calculated for both visually assessed disease traits and the VIs taken at different time points throughout the growing season. The *H*^2^ of the visual scores and VIs show a gradual increase throughout the growing season peaking at R8 (harvest maturity). RGB indices Intensity and NDLuv had *H*^2^ = 0 throughout the growing season. The *H*^2^ of the visually assessed LLS scores was higher than that of both GRD incidence and severity throughout the season. The *H*^2^ of the VIs was highest at R8 with exception of CSI which was peaked at R4 (pod formation). The heritability of LLS scores was higher compared to any associated VI at all times of data collection. The strongly associated VIs at R8 [NDVI (*H*^2^ = 0.87), v*(*H*^2^ = 0.72), and b*(*H*^2^ = 0.60)] and R4 (CSI, *H^2^* = 0.75) equally had a high heritability. The *H*^2^ of the GRD visual scores was lower than associated indices; However, all VIs strongly associated with GRD visual scores [NDVI (*H*^2^ = 0.87), v* (*H*^2^ = 0.72), Hue (*H*^2^ = 0.81), GA (*H*^2^ = 0.77), and GGA (*H*^2^ = 0.80)] at R8, and CSI (*H^2^* = 0.64) and NDVI (*H*^2^ = 0.86) at R7 (beginning of maturity) all had a higher heritability compared to the GRD visual scores.

**TABLE 6:** Broad-sense heritability of HTP derived indices and visual scores for late leaf spot (LLS) and groundnut rosette virus (GRD) during the 2020A and 2020B growing seasons at different groundnut growth stages.

	Phenological stage			
Trait	R2	R4	R7	R8
NDVI	0.30	0.00	0.86	0.87
Intensity	0.00	0.00	0.00	0.00
Hue	0.54	0.17	0.43	0.81
Saturation	0.58	0.41	0.00	0.20
Lightness	0.00	0.02	0.00	0.54
a*	0.48	0.14	0.53	0.73
b*	0.55	0.32	0.00	0.60
ab	0.41	0.11	0.48	0.68
NDLab	0.29	0.17	0.11	0.34
u*	0.35	0.16	0.51	0.68
v*	0.56	0.27	0.00	0.72
uv	0.00	0.15	0.50	0.64
NDLuv	0.00	0.00	0.00	0.00
GA	0.49	0.09	0.58	0.77
GGA	0.43	0.06	0.64	0.80
CSI	0.39	0.75	0.64	0.66
CT	0.06	0.06	0.00	0.50
**Visual score**				
LLS	0.87	0.87	0.93	0.95
GRD incidence	0.25	0.25	0.44	0.37
GRD severity			0.43	0.43

*In this case is associated with the CIE-Lab indices a and b and CIE-Luv indices u and v as derived from the BreedPix.

### Ordinal Logistic Models to Predict Late Leaf Spot and Groundnut Rosette Disease Severity

Late leaf spot severity was scored on a 1–9 scale, however, at R8 (harvest maturity), 6 levels were present and scores were between 4 and 9. The probability of predicting the LLS VI-derived score P_4_ + P_5_ + P_6_ + P_7_ + P_8_ + P_9_ = 1. The model for LLS predicted scores is presented below;


$$P_{4}=\frac{1}{1+e^{\left(-5.78-\beta \right)}}$$

$$P_{5}=\frac{1}{1+e^{\left(-4.17-\beta \right)}}-P_{4}$$

$$P_{6}=\frac{1}{1+e^{\left(-0.36-\beta \right)}}-P_{4}-P_{5}$$

$$P_{7}=\frac{1}{1+e^{\left(-1.5-\beta \right)}}-P_{4}-P_{5}-P_{6}$$

$$P_{8}=\frac{1}{1+e^{\left(-3.72-\beta \right)}}-P_{4}-P_{5}-P_{6}-P_{7}$$

$$P_{9}=1-P_{4}-P_{5}-P_{6}-P_{7}-P_{8}$$

Where *e* = 2.718 is the Euler’s number,

β = –1.81NDVI + 0.88CSI–2.2b*.

The LLS model with NDVI and RGB indices CSI and b* as the best predictors had AIC of 431.94 and BIC of 463.53 compared to the full model (with all the predictors) with AIC of 492.014and BIC of 520.09. The model had a kappa value of 0.52 and an overall accuracy of 64% (Table 7). The model had the highest predictability for the visual scores 6 and 9, and the lowest prediction accuracy for the visual score 4. The specificity of the model was high with the lowest value being 0.83 for visual score 6. The highest misclassification of scores was observed at visual score 7 with 9 scores allocated to score 6 by the model. The prediction accuracy of the model increased to 91% when the nearest score method was used.

**TABLE 7:** Classification confusion matrix of LLS visual scores and model predicted scores of the training data set collected at Nakabango.

		Predicted score					
Breeder score	Plots (n)	4	5	6	7	8	9
4	16	**3**	1	12	0	0	0
5	29	2	**7**	20	0	0	0
6	82	1	4	**69**	3	2	3
7	31	0	0	12	**12**	5	2
8	34	0	0	1	6	**17**	10
9	55	0	0	1	0	5	**49**
**Total**	247						
Accuracy	64%	19%	24%	84%	39%	50%	89%
Nearest score	91%						

*The bold figures represent the visual scores which were correctly classified by logistic models.*

The Groundnut rosette disease severity was scored on a 1–5 scale. The probability of predicting GRD VI-derived score was P_1_ + P_2_ + P_3_ + P_4_ + P_5_ = 1. The model for GRD predicted is presented below;

$$P_{1}=\frac{1}{1+e^{\left(-0.98-\beta \right)}}$$

$$P_{2}=\frac{1}{1+e^{\left(-0.33-\beta \right)}}-P_{1}$$

$$P_{3}=\frac{1}{1+e^{\left(-1.12-\beta \right)}}-P_{1}-P_{2}$$

$$P_{4}=\frac{1}{1+e^{\left(-1.77-\beta \right)}}-P_{1}-P_{2}-P_{3}$$

$$P_{5}=1-P_{1}-P_{2}-P_{3}-P_{4}$$

Where *e* = 2.718 is the Euler’s number,

β = 0.044Hue + 0.42a*–0.006*uv*

The GRD model with RGB indices Hue, a*, and uv selected as the best predictors of GRD severity; this model had AIC of 619.2 and BIC of 643.8 compared to the full model (with all predictors) with AIC of 626.5 and BIC of 700.3. The model had a kappa of 0.28 and overall model accuracy of 45%. The model had the highest predictability for the visual scores of 5 followed by 2 and 1. The lowest prediction accuracy was recorded for the class 4. The misclassification between classes was highest between 1 and 2. Twenty-four plots visually scored 1 were given a score of 2 by the model. Similarly, 26 plots which were scored as 2, were predicted by the model as 1 (Table 8). The lowest misclassification was for 4 as indicated by the specificity of 1.00. The prediction accuracy of the model however increased to 84% when the nearest score method was used.

**TABLE 8:** Confusion matrix of GRD visual scores and the model predicted scores of the training dataset of the data collected at Nakabango.

		Predicted score				
Breeder score	Plots (n)	1	2	3	4	5
1	58	**31**	24	2	0	1
2	80	26	**43**	6	0	5
3	45	1	20	**12**	0	12
4	30	1	7	10	**0**	12
5	35	0	2	8	0	**25**
**Total**	248					
Accuracy	45%	53%	54%	24%	0%	71%
Nearest score	84%					

*The bold figures represent the visual scores which were correctly classified by logistic models.*

### Validation of the Regression Models

The LLS logistic model was validated using the data collected at NaSARRI 2020B. The model performed with lower accuracy of 40% but performed with a similar accuracy when the nearest score accuracy was used with 88% (Table 9). The Pearson correlation between the model derived score and the breeder score of the validation data was moderate (*r* = 0.58). The GRD logistic model performed with similar accuracy with the validation data set with 38 and 91% (Table 10) when the nearest accuracy was used.

**TABLE 9:** Confusion matrix of LLS visual scores of the breeder scores and predicted scores of data collected at NaSARRI 2020B.

		Predicted score					
Breeder score	Plots (n)	4	5	6	7	8	9
4	0	**0**	0	0	0	0	0
5	3	0	**0**	3	0	0	0
6	52	0	0	**21**	24	6	1
7	26	0	0	6	**15**	3	2
8	30	0	0	0	11	**15**	4
9	38	0	0	3	6	20	**9**
**Total**	149						
Accuracy	40%	0%	0%	40%	58%	50%	24%
Nearest score	88%						

*The bold figures represent the visual scores which were correctly classified by logistic models.*

**TABLE 10:** Confusion matrix of GRD severity visual scores and the model predicted scores of data collected at NaSARRI 2020A and 2020B.

		Predicted score				
Breeder score	Plots scored	1	2	3	4	5
1	135	**25**	97	12	0	1
2	149	23	**92**	32	0	2
3	46	2	28	**15**	0	1
4	15	0	11	4	**0**	0
5	1	0	0	1	0	**0**
**Total**	346					
Accuracy	38%	19%	62%	33%	0%	0%
Nearest score	91%					

*The bold figures represent the visual scores which were correctly classified by logistic models.*

## Discussion

### Association Between Visual Scores and High-Throughput Phenotyping Derived Indices

**FIGURE 6:**
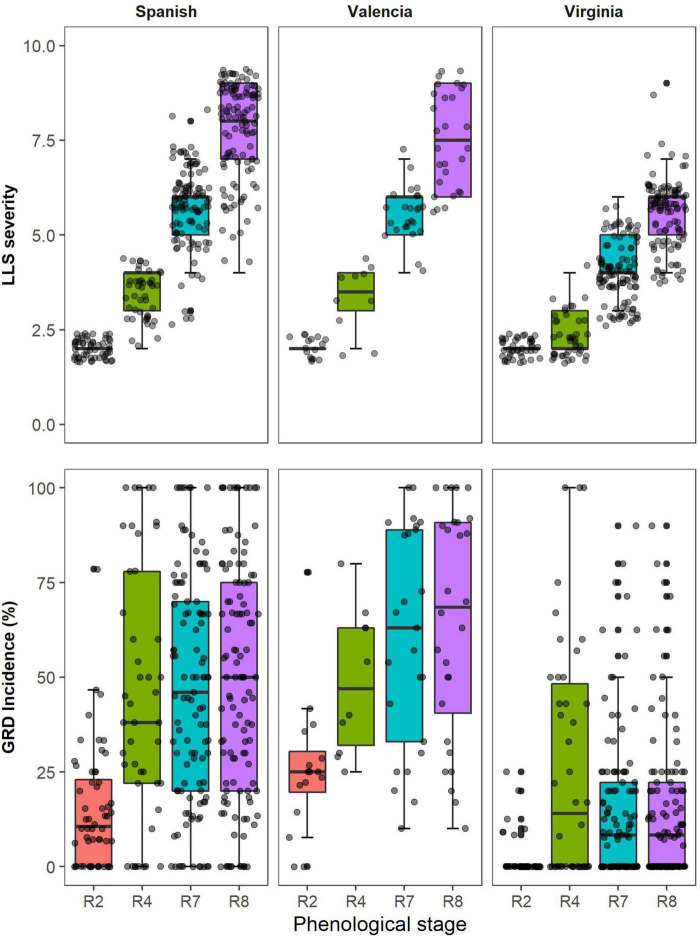
Distribution of visual scores of Late leaf spot (LLS) severity and Groundnut rosette disease (GRD) incidence for genotypes belonging to three groundnut market types (Spanish, Valencia, and Virginia) at different phenological stages within the season.

In this study, the VIs were significantly correlated with the visual scores of groundnut LLS severity among different groundnut genotypes and market groups, and at different growth stages. Overall, NDVI had the strongest relationship with visual symptoms of the different market types at harvest maturity. NDVI had a negative relationship with LLS severity, i.e., more severe LLS disease less NDVI values, and the effect of the disease was evident at full pod formation (R4) for Spanish. There was a large variation among the Spanish and Valencia groups because the susceptible genotypes were easily affected by the LLS symptoms compared to the Virginia groundnuts which had a low variation of disease symptoms at this stage (Figure 6). The correlations were stronger for all market types (*r* = –0.85) harvest maturity, because there was a higher disease variation and a clear distinction between resistant and susceptible genotypes (Figure 4). Moderate correlations between LLS and NDVI were observed among the Virginia market type (*r* = –0.64) compared to the Spanish (*r* = –0.89) and Valencia (*r* = –0.83) because 75% of the genotypes had scored less than 5 hence belonging to the resistant group (Figure 6). As shown by others, higher NDVI values were associated with healthy canopies (in this case, resistant genotypes) with the ability to absorb red light and effectively reflect NIR (Liu and Huete, 1995; Govaerts and Verhulst, 2010). Increased LLS severity is characterized by increased defoliation (Subrahmanyam et al., 1995) and reduction of leaf chlorophyll content (Singh et al., 2011), thus reducing the light-absorbing capacity of canopies for the susceptible genotypes. Chlorophyll, which is responsible for light absorption, is linearly related to NDVI (Gitelson and Merzlyak, 1996). The reduction in reflectance of the near-infrared radiation is due to the reduction in multiple leaf layers within the canopy, and the increase in background exposure (Knipling, 1970) therefore, reducing NDVI with increase in severity.

Several RGB indices were strongly correlated with visual LLS symptoms (Figure 4). Hue, GA, and Saturation (*r* = –0.55, –0.68, –0.66) were moderately correlated with visual scores for all groundnut market types at harvest maturity (R8). The effect of LLS on canopy CSI was apparent as early as R4 (at full pod formation) but the effects on hue and GA became visible at R7 (beginning of maturity) when there was clear variation among genotypes. The change of the correlation signs of CSI from negative at beginning of peg formation (R2) to positive at beginning of pod formation indicated the effect of the disease on the canopy color and leaf senescence, i.e. at peg formation plants were small and, in absence of disease, other factors influenced senescence; at pod formation disease severity doubled and this was the main factor for increased crop senescence. Lower hue and GA values among susceptible genotypes could be attributed to increased yellowing of the canopy due to a decrease in leaf chlorophyll (Sarkar et al., 2021). Defoliation of leaves could also lead to reduced hue and GA since both are associated with presence of green biomass (Casadesús et al., 2007). In highly defoliated canopies (as evident in susceptible genotypes), the soil background is not completely covered. The hue of bare soil is low (Zhou et al., 2015) therefore, the hue of highly defoliated canopies (susceptible) is lower than that in well-developed canopies (resistant genotypes). The correlation among Virginia groundnuts was lower for both GA (*r* = –0.3) and hue (*r* = –0.04) because of the low variation of both, visual scores and VIs (Figure 4). b* (*r* = –0.75) and v*(*r* = –0.8) were highly correlated with visual scores of LLS among all market types. This is attributed to the yellowing of the canopy (Serret et al., 2020) due to reduced leaf chlorophyll (Singh et al., 2011; Sarkar et al., 2021). Although these indices were able to identify the canopy yellowing later in the season, CSI (*r* = 0.76) identified yellowing of the canopies due to LLS as early as the pod formation. Early detection of LLS would aid early selection of resistant genotypes saving resources in the breeding program. Canopy temperature (*r* = 0.42), was however moderately correlated with the visual scores. Canopy temperature is associated with transpirational cooling of the plant canopy (Pilon et al., 2018). Cool plant canopies are associated with healthy canopies. An increase in canopy temperature is associated with an increase in disease severity (Oerke et al., 2006). This could be attributed to the reduction of evaporative surfaces due to defoliation and damage of leaf surfaces by leaf spot lesions. Results in our study indicated that LLS severity was promoted by warmer canopy temperatures.

Several RGB indices demonstrated moderate to high correlations with GRD visual scores with different market types at different weeks after planting (Figure 5). Generally, the RGB indices Hue, GA, GGA, v*, and b* were negatively correlated with GRD symptoms. GRD is characterized by shortening of stem internodes, rosetting of the leaves, chlorosis of leaves, and overall stunting of the affected plants (Table 3; Naidu et al., 1998; Waliyar et al., 2007). Indices such as Hue, GA, and GGA which are associated with the presence of green biomass (Casadesús et al., 2007; Casadesús and Villegas, 2014), were, therefore, able to differentiate the resistant and susceptible genotypes based on the size of the plant. Healthy plants have larger canopies compared to the stunted rosette plants, thereby occupying more green area and consequently covering more soil background. Since hue angle is also affected by soil background which has a lower hue value (Zhou et al., 2015), healthy genotypes have a higher hue angle compared to the stunted susceptible genotypes. Increased yellowing of susceptible genotypes was observed. This was evident from the reduction of GA and GGA pixels, and the increase of b* and v*. Yellowing of leaves has been associated with chlorophyll reduction due to drought in various crops (Serret et al., 2020; Sarkar et al., 2021); in our case, this was due to the high prevalence of chlorotic rosette symptoms (Naidu et al., 1998) also known as yellow rosette symptoms, for which Nakabango is well known as a hotspot (Okello et al., 2014). The chlorotic symptoms were visible early in the season by during the beginning flowering stage and later in the season, toward harvest. NDVI (*r* = –0.72), which has widely been used for monitoring canopy health status was also negatively correlated with the GRD symptoms. NDVI is indicative of the amount biomass and vigor of vegetation within the plot, and therefore plant biomass (Cabrera-Bosquet et al., 2011). In our case, the reduction of NDVI among susceptible genotypes could be due to reduction of the biomass, vigor, and leaf chlorophyll content. Canopy temperature increased with an increase in GRD severity although the correlation was weak (*r* = 0.42) at beginning of maturity. GRD is associated with damage of the leaf xylem (Favali, 1977) and this could limit the water transport to the leaves hence causing reduced transpirational cooling. The findings in this study support our hypothesis that there are HTP-derived indices that are correlated with visual scores of LLS and GRD symptoms. Therefore, indices which are highly correlated with LLS and GRD scores can be applied as secondary traits for indirect selection for disease resistance.

### Broad-Sense Heritability

Although the visual scores were highly correlated to several VIs, this alone is not enough to justify replacement of visual scores. Broad sense heritability (*H*^2^), also known as repeatability (Piepho and Mohring, 2007) was measured for each of the sensor-derived indices taken at the same time as the visual scores. The measurement of *H*^2^ using different methods (HTP-derived traits and visual scores) could be an estimate of measurement error since the genotypes are constant and environmental effect should be negligible (Piepho and Mohring, 2007; Crain et al., 2016). A high *H*^2^ is indicative of the precision and predictive ability for the secondary traits associated with LLS and GRD resistance (Crain et al., 2016, 2018). Several VIs such as NDVI, GA, GGA, and v* which were strongly associated with the disease visual scores had comparable or higher *H*^2^ making them suitable for selection. The high heritability is attributed to high genetic variation which is essential for genetic gain in breeding (Table 6). High NDVI *H*^2^ values have also been reported in other studies in cotton (0.28–0.9) and wheat (Andrade-Sanchez et al., 2013; Crain et al., 2016). The high *H*^2^ could indicate the consistence of the measurements across the different environments (Andrade-Sanchez et al., 2013). The visual scores of LLS had high heritability (*H*^2^ = 0.85) as early as peg formation. This can be attributed to the high variation within the population for LLS resistance. Similar results were reported for *H*^2^ for LLS resistance based on leaf defoliation by Anderson et al. (1991). In contrast to LLS, the broad-sense heritability of GRD was lower compared to those in previous studies (*H*^2^ = 0.74) (Merwe et al., 1999). This could be attributed to the low variation of VIs at that time of the season (De Swaef et al., 2021). Therefore, NDVI, hue, v*, CSI, GA, and GGA which were highly correlated with the visual scores and had high *H*^2^ can be applied for indirect selection for LLS and GRD resistance in groundnut breeding, if timing is toward later part of the season. These findings confirm our hypothesis that HTP derived indices are highly heritable.

### Ordinal Logistic Models

Disease classification models developed in this study were effective in distinguishing disease symptoms. The LLS and GRD models had classification accuracies of 64 and 45% respectively. The LLS select model contained NDVI, CSI, and b* indices. The NDVI in the model is associated with canopy biomass (Cabrera-Bosquet et al., 2011) and canopy health (Kefauver et al., 2015) which are both affected by LLS symptoms. The CSI and b* represent canopy color changes from green to yellow (Zaman-Allah et al., 2015). This is associated with chlorophyll detoriation among susceptible genotypes (Singh et al., 2011). The GRD model contained RGB indices hue, a*, and *uv* which represent canopy color. These indices are associated with the presence of green biomass within the plot (Serret et al., 2020). Hue and a* represent the greenness of the canopy while *uv* represents both the green and yellow part of the canopy (De Swaef et al., 2021), which could include the green and chlorotic symptoms of GRD in our case (Naidu et al., 1998; Waliyar et al., 2007; Okello et al., 2014). The accuracy of the models increased to 91 and 84% for LLS (Table 7) and GRD (Table 8) classification models, respectively. The nearest score method takes into account that different evaluators, or the same evaluator could rate the same plot differently depending on the time of the day and number of plots scored (Sarkar et al., 2021). For example, on the 1–9 LLS scale, a score of 6 could be under scored as a 5 or over scored as a 7. Similarly, on 1–5 GRD severity scale, a score of 2 could be scored as a 1 or a 3. The method assumes that the model can classify a plot as 1 or 3 instead of a 2 because the reflectance from the plot could be closer to that from a plot scored as a 1 or a 3. This method is the closet to the visual scores by the breeder also gives the because it takes into account that the model is trained based on the visual scores which are not perfect. This misclassification could also be attributed to the differences among the groundnut market types; the three market types have different leaf color, plant height and canopy architecture. These small differences could affect the visual score from one plot to another. The nearest scores method has a higher accuracy and it could be actively applied for selection for LLS and GRD resistance since it increases chances of selecting for genotypes of interest. For example, when selecting for resistance for GRD, a cut off is set at score of 2 (resistant). When the nearest score method is applied, there are higher chances of selecting a score of 1 (highly resistant) or 3 (moderately resistant) (Table 3). The logistic models developed in this study had high accuracy using the nearest score method when applied to independent data sets (Tables 9, 10) indicating the effectiveness of the models and method for selection for resistance in different environments. The findings of this study affirm our hypothesis that VI-derived models can be consistent for routine selection for resistance in breeding programs.

## Conclusion

Results from this work present novel methods of screening for LLS and GRD resistance using HTP derived VIs. These results illustrate that HTP derived indices from handheld sensors such as the RGB camera, GreenSeeker, and the thermal camera can complement or even act as alternatives to the visual scores used by breeders. These novel methods have the potential to enhance faster and reduced cost development of new varieties. However, for such methods to adopted by breeders for active deployment in selection, they have to be highly automated to eliminate drudgery. The application of individual sensors reduced the data collection time by almost half, but the cumulative time spent using the three different sensors was almost the same as that spent when using conventional methods. Therefore, these methods did not meet the desired throughput and might not be feasible in cases where the experiments are very large. Nonetheless, there is potential to improve the methods described in this study. More efficient methods of data collection such as use of unmanned aerial vehicles (UAVs). Recent advances in plant phenotyping involve the use of unmanned aerial vehicles (UAVs) to collect several images generating large amounts of data. Several studies have reported that UAVs are faster and more effective for phenotyping large populations for traits such as height and drought tolerance in groundnut breeding (Sarkar et al., 2020, 2021) hence providing the desired high-throughput. This study therefore lays the foundation for investment in such more advanced equipment in groundnut breeding for selection for resistance to late leaf spot and groundnut rosette disease which are the most important foliar diseases in Uganda and SSA.

## Data Availability Statement

The original contributions presented in the study are included in the article/Supplementary Material, further inquiries can be directed to the corresponding author.

## Author Contributions

MB wrote the proposal and won the grant under the PIL. DO selected the study materials. IC and DO designed the experiments. IC took the field measurements, carried out the data analysis with assistance from SS, RO, and TO, and wrote the manuscript under the supervision of DO, RO, and MB contributed to the editing and reviewing of the manuscript. All authors contributed to the article and approved the submitted version.

## Author Disclaimer

The contents are the responsibility of the authors and do not necessarily reflect the views of USAID or the United States Government.

## Conflict of Interest

The authors declare that the research was conducted in the absence of any commercial or financial relationships that could be construed as a potential conflict of interest.

## Publisher’s Note

All claims expressed in this article are solely those of the authors and do not necessarily represent those of their affiliated organizations, or those of the publisher, the editors and the reviewers. Any product that may be evaluated in this article, or claim that may be made by its manufacturer, is not guaranteed or endorsed by the publisher.

## Footnotes

1
https://www.bmspro.io

2
http://github.com/george-haddad/CIMMYT

3
http://fiji.sc/Fiji
